# Emergence of *mutH*^V76G^ in carbapenem-resistant *Klebsiella pneumoniae* disrupts DNA mismatch repair and results in a hypermutator phenotype

**DOI:** 10.1128/aac.00189-26

**Published:** 2026-08-05

**Authors:** Shaoji Cheng, Cornelius J. Clancy, Giuseppe Fleres, Hassan Badrane, Matthew J. Culyba, Anthony Newbrough, Liang Chen, M. Hong Nguyen

**Affiliations:** 1 Department of Medicine, University of Pittsburgh 199716, Pittsburgh, Pennsylvania, USA; 2 Department of Pharmacy and Pharmaceutical Sciences, School of Pharmacy and Pharmaceutical Sciences, University at Buffalo 15497, Buffalo, New York, USA; Entasis, Big Bay, Michigan, USA

**Keywords:** hypermutator, DNA mismatch protein, MutH, carbapenem-resistant, *Klebsiella pneumoniae*

## Abstract

Hypermutation-driven evolution is a major contributor to antibiotic resistance in some bacterial pathogens, but its role in *Klebsiella pneumoniae* remains poorly defined. We analyzed 11 KPC-producing ST258 *K. pneumoniae* isolates collected serially over ~4 years from a host with persistent colonization and recurrent infections. After >3 years, isolates acquired ceftazidime–avibactam (CZA) resistance with restored carbapenem susceptibility, coinciding with emergence of a V76G substitution in MutH, a conserved endonuclease in the DNA mismatch repair pathway. Isolates carrying *mutH^V76G^* demonstrated sharp increases in within-host genetic diversification (69–179 versus 2–12 SNPs), and accumulated mutations across multiple resistance-associated loci, including *bla*_KPC-3_, *ompK36*, *cirA*, and *envZ*. Both clinical *mutH^V76G^* isolates and CRISPR-Cas9-engineered mutants (*mutH*^V76G^ and *mutH* null mutant) exhibited hypermutator phenotypes and showed accelerated acquisition of resistance or reduced susceptibility to CZA, meropenem–vaborbactam (MVB), and cefiderocol. Using matched isogenic engineered strains, we confirmed that *mutH^V76G^* heightens the pace of resistance evolution *in vitro*, enhances plasmid uptake and transfer, and increases bacterial fitness during mouse infections. Resistance pathways that emerged *in vivo* paralleled those observed clinically, including *bla*_KPC-3_ variants under CZA pressure and *ompK36* mutations under MVB exposure. The similarity of *mutH^V76G^* and *mutH*-null phenotypes indicates that the V76G substitution largely abolishes MutH function. These findings identify MutH-mediated hypermutation as an adaptive strategy in *K. pneumoniae* that accelerates resistance to multiple last-line antibiotics and promotes horizontal gene transfer without apparent fitness cost.

**IMPORTANCE:**

Antibiotic-resistant *Klebsiella pneumoniae* is a major global health threat, and resistance to new “last-line” antibiotics is rising. In this study, we examined a rare collection of carbapenem-resistant *K. pneumoniae* isolates obtained over 4 years from a single host, giving us an opportunity to observe how the bacterium evolved over time. During this period, the bacteria acquired a single nucleotide change in a DNA damage repair gene *mutH*, which caused them to accumulate mutations far more rapidly than with wild-type *mutH*. Through extensive genomic and experimental work, we found that this single change produced a hypermutator strain capable of quickly developing resistance to several key antibiotics, including ceftazidime–avibactam and meropenem–vaborbactam, and also reduced susceptibility to cefiderocol. Using laboratory-engineered strains, animal infection models, and detailed genetic analyses, we confirmed that the *mutH* mutation accelerates resistance development and increases the bacterium’s ability to acquire resistance plasmids. Importantly, the same types of mutations that appeared under laboratory conditions also emerged during infection. Our findings show how hypermutation can compromise even the newest antibiotics and highlight the need for surveillance of DNA repair defects in antibiotic-resistant *K. pneumoniae*.

## INTRODUCTION

Carbapenem-resistant Enterobacterales (CRE) present an urgent threat to global public health. *Klebsiella pneumoniae* is a leading cause of CRE infections in the United States, in particular infections caused by carbapenemase-producing isolates (1). CR-*K. pneumoniae* (CRKP) infections are associated with high morbidity and mortality. Over the past decade, novel β-lactam–β-lactamase inhibitor combinations, such as ceftazidime–avibactam (CZA) and meropenem–vaborbactam (MVB), have improved outcomes in serious CRE infections (2–5). However, resistance to these agents develops in a subset of patients (~5%–8%), frequently driven by mutations in the KPC Ω-loop or OmpK36 porin (3, 6). Chronic gastrointestinal (GI) colonization and recurrent invasive infections, typically originating from the GI reservoir, are common among survivors of CRE infections (7–9). There is a need to better understand mechanisms underlying within-host evolution and adaptation of CRKP and other CREs.

A small fraction of bacterial populations (~1%–5%) exhibits elevated spontaneous mutation rates, known as hypermutation (10). Hypermutator phenotypes are particularly prevalent during chronic infections by bacteria, such as *Pseudomonas aeruginosa* and *Staphylococcus aureus* (11–13). Hypermutation is most often caused by inactivation of methyl-directed DNA mismatch repair (MMR) (14). In Enterobacterales, MutS, MutL, and MutH proteins coordinate MMR (15). MutS recognizes and binds to DNA mismatches and recruits MutL. The MutS-MutL-DNA complex, in turn, binds to and activates the latent endonuclease activity of MutH, which is bound to DNA at a neighboring *dam* methylation site. MutH specifically nicks the newly synthesized (unmethylated) DNA strand at the mismatched site, thereby marking the strand containing the erroneously incorporated nucleotide for excisional repair (14, 16, 17). This process is critical for maintaining high-fidelity DNA replication. In *Escherichia coli*, loss of function in any of these Mut proteins produces a fixed hypermutator phenotype, which can accelerate bacterial adaptation under selective pressures, including the acquisition of antibiotic resistance through point mutations or plasmid-mediated gene transfer (18–21). Despite its potential clinical significance, hypermutation has been poorly characterized in CRKP and other CREs (22–25).

In this study, we leveraged a rare, longitudinal collection of 11 KPC-producing *K. pneumoniae* isolates spanning over 4 years from an immunocompromised patient with chronic GI colonization and recurrent invasive infections. Whole genome sequencing (WGS) revealed the emergence of a *mutH* point mutation during CZA treatment, which conferred a V76G substitution in a conserved region of MutH that is important for endonuclease activity. Using both clinical and engineered *mutH* mutant strains in *in vitro* and mouse experiments, we demonstrate that *mutH*^V76G^ induced a hypermutator phenotype, drove rapid genomic diversification (including acquisition of *bla*_KPC_ mutations conferring CZA resistance and restoring meropenem susceptibility), enhanced horizontal plasmid transfer, and increased bacterial fitness *in vivo*. Our study provides an unprecedented longitudinal view of CRKP evolution in the human host and demonstrates how hypermutation can act as a potent mediator of multidrug resistance and adaptive traits relevant to colonization and infection.

## RESULTS

### Longitudinal *K. pneumoniae* isolates reveal emergence of *mutH*^V76G^ and CZA resistance

**Fig 1:**
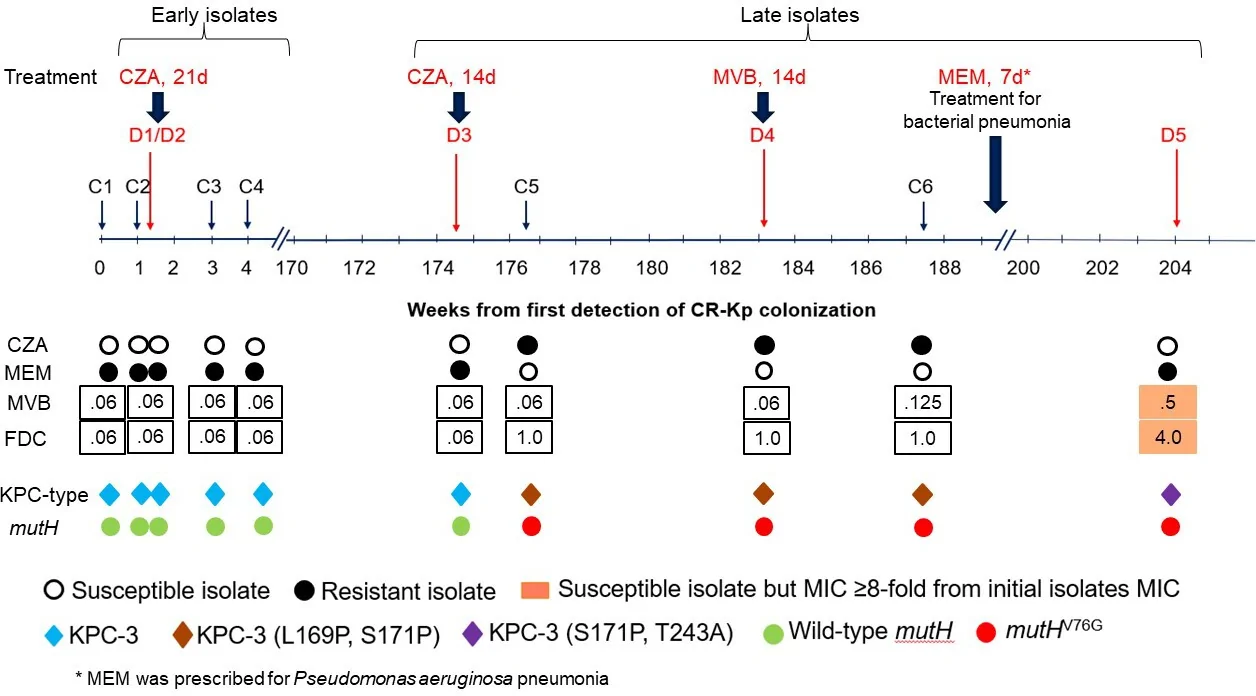
Timeline and features of longitudinal *Klebsiella pneumoniae* isolates. Eleven isolates collected from a lung transplant recipient are included in this study. Six early isolates (C1, C2, D1, D2, C3, C4) were recovered within a 4-week period following lung transplantation. Five late isolates (D3, C5, D4, C6, D5) were recovered >3 years after C1. Isolates with names prefixed by C (colonization) were recovered from rectal surveillance cultures. Isolates with names prefixed by D were cultured from various sites of suspected disease. Antibiotic treatment regimens are shown above the timeline (start dates noted by black arrows). Antibiotic susceptibility, KPC type and *mutH* genotype are shown below the timeline. MVB and FDC MICs are presented as µg/mL. All isolates were MVB and FDC susceptible, but MICs were 4–64-fold higher against the last isolate (D5) than they were against earlier isolates.

We analyzed 11 longitudinal KPC-producing *K. pneumoniae* isolates collected from sites of colonization (C) or invasive disease (D) over 4 years. Isolates were categorized into early and late groups (Fig. 1). Early isolates (collected within 4 weeks after lung transplantation) included C1 and C2 (i.e., first and second colonization isolates, recovered from serial surveillance rectal swabs), D1 and D2 (i.e., first and second disease isolates associated with pneumonia and empyema, respectively), and C3 and C4 (surveillance rectal swabs collected after D2). Late isolates (collected between 175 weeks [~3.3 years] and 204 weeks [~3.9 years] after C1) were D3 (bacteremia), C5 (rectal surveillance), D4 (tracheal aspirate), C6 (rectal surveillance), and D5 (empyema). All isolates belonged to sequence type 258 (ST258) lineage and carried *bla*_KPC_ genes.

Early isolates and the first late isolate (D3) were meropenem (MEM)-resistant (MIC >16 µg/mL) and CZA-susceptible (MIC = 0.125 µg/mL), and harbored *bla*_KPC-3_ and wild-type *mutH*. Following a 2-week CZA treatment course for D3 bacteremia, late isolates C5, D4, and C6 were CZA-resistant and MEM-susceptible. These isolates carried a *mutH* point mutation encoding a V76G substitution (*mutH*^V76G^) and accumulated KPC Ω-loop mutations (L169P, S171P) (Fig. 1; Fig. S1). *bla*_KPC-3_^L169P^ is known to confer CZA resistance and restore MEM susceptibility (24). The final longitudinal isolate (D5) reverted to MEM resistance and CZA susceptibility, maintained *mutH*^V76G^, lost *bla*_KPC-3_^L169P^, and acquired *bla*_KPC-3_^T243A^.

All 11 isolates were susceptible to cefiderocol (FDC) and MVB. FDC MICs increased from 0.06 µg/mL in early isolates and D3 to 1.0 µg/mL in later isolates (C5, D4 and C6). In the final isolate (D5), the FDC MIC further increased to 4.0 µg/mL. Similarly, MVB MIC increased from 0.06 µg/mL in early isolates, D3, C5, and D4 to 0.125 µg/mL in C6 to 0.5 µg/mL in D5 (Table 1). Late isolates C5 through D5 acquired mutations in *envZ*, which encodes the kinase of a two-component system involved in iron transport and regulation of outer membrane porins (*envZ*^L19P^ in all isolates; *envZ*^K358I^ also present in C5 and D4). In addition, D5 uniquely acquired porin *ompK36*^Q301W^ and iron transporter *cirA*^Y294C^ mutations. No coding sequence differences were detected in penicillin-binding protein gene *PBP3*, regulatory genes (*baeR, ompR*), siderophore uptake genes (*fepA, fhuA, tonB*), or RND efflux pump components (*acrA, acrB, tolC, mexB, oprM*) (Table 1). These findings do not exclude potential contributions from changes in gene expression, regulatory variation outside the loci examined, or other non-coding mechanisms.

**TABLE 1:** Evolution of antibiotic MICs and susceptibility among longitudinal *K. pneumoniae* isolates

Isolates	MEM, CZA, MVB susceptibility and MICs	FDC MIC^*a*^	KPC-3 mutations	OmpK36 mutations	CirA mutations	EnvZ mutations
C1, C2, D1, D2, C3, C4 (early isolates)	MEM resistant (MIC > 16 µg/mL) CZA susceptible (MIC = 0.125/4 µg/mL) MVB susceptible (MIC = 0.06/8 µg/mL)	0.125 µg/mL	Wild type	Wild type	Wild type	Wild type
D3 (late isolate, pre-treatment)	MEM resistant (MIC > 16 µg/mL) CZA susceptible (MIC = 0.125/4 µg/mL) MVB susceptible (MIC = 0.06/8 µg/mL)	0.06 µg/mL	Wild type	Wild type	Wild type	Wild type
C5, D4, C6 (late isolates, post-treatment)	MEM susceptible (MIC = 0.06 µg/mL) CZA resistant (MIC ≥ 16/4 µg/mL) MVB susceptible (C5, D4 MIC = 0.06/8 µg/mL; C6 MIC = 0.125/8 µg/mL)	1 µg/mL	L169P, S171P	Wild type	Wild type	L19P, K358I (C5, D4) L19P (C6)
D5 (last isolate)	MEM resistant (MIC ≥ 64 µg/mL) CZA susceptible MVB susceptible (MIC = 0.5/8 µg/mL)	4 µg/mL	S171P, T243A	Q301W	Y294C	L19P, K358I

^*a*^ Cefiderocol MICs were increased against C5, D4, C6, and D5 but remained within susceptible range. *ompK36*^Q301W^, *cirA*^Y294C^, *envZ*^L19P^, and *envZ*^K358I ^substitutions are observed uniquely in C5, D4, C6, and D5; their functional contributions to increased FDC MICs has not been experimentally confirmed.

**Fig 2:**
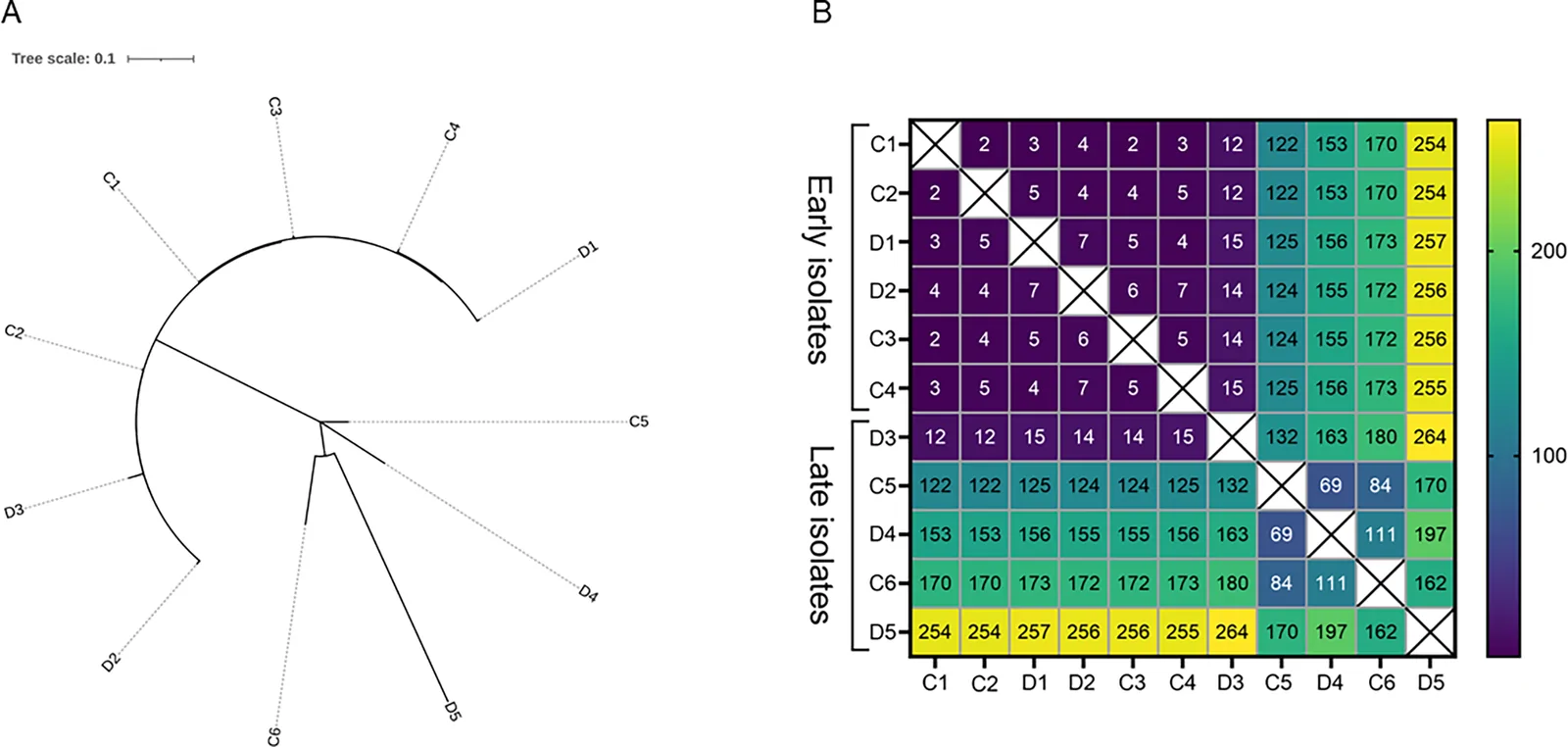
Core genome single nucleotide polymorphism (SNP) phylogeny and SNP distance matrix for longitudinal *K. pneumoniae* isolates. (**A**) Core genome SNP-based maximum likelihood phylogenetic tree. Evolutionary relationships are shown for 11 longitudinal KPC-producing *K. pneumoniae* isolates collected from a lung transplant recipient over 4 years. Early isolates (C1, C2, D1, D2, C3, C4) clustered closely with the first late isolate (D3), whereas subsequent late isolates (C5, D4, C6, D5) were progressively more divergent. The tree was built from an alignment of core genome SNPs, with bootstrap support of 100% for every cluster. (**B**) Pairwise SNP distance matrix. The table gives the number of SNP differences between isolates. The color gradient reflects genomic divergence, with dark purple indicating closely related isolates (low SNP distances) and yellow indicating greater divergence (high SNP distances). Early isolates (C1, C2, D1, D2, C3, C4) and the first late isolate (D3) were highly similar, differing by only 2–15 SNPs. In contrast, later isolates carrying *mutH^V76G^* (C5, D4, C6, D5) showed progressively greater divergence from the earlier cluster (122–264 SNPs) and from one another (69–197 SNPs), consistent with a hypermutator phenotype driving accelerated within-host evolution.

Core genome SNP analysis revealed that early isolates were highly clonal, differing by only 2–7 SNPs. The first late isolate (D3) remained closely related to this early group, with 12–15 SNP differences (Fig. 2A). In contrast, late mutHV76G isolates (C5, D4, C6, D5) diverged substantially (122–264 SNPs vs early isolates; 69–197 SNPs among themselves) (Fig. 2B). Maximum likelihood phylogeny confirmed that *mutH*^V76G^ acquisition coincided with accelerated within-host genomic diversification.

### *mutH*^V76G^ disrupts a conserved motif and confers hypermutation

**Fig 3:**
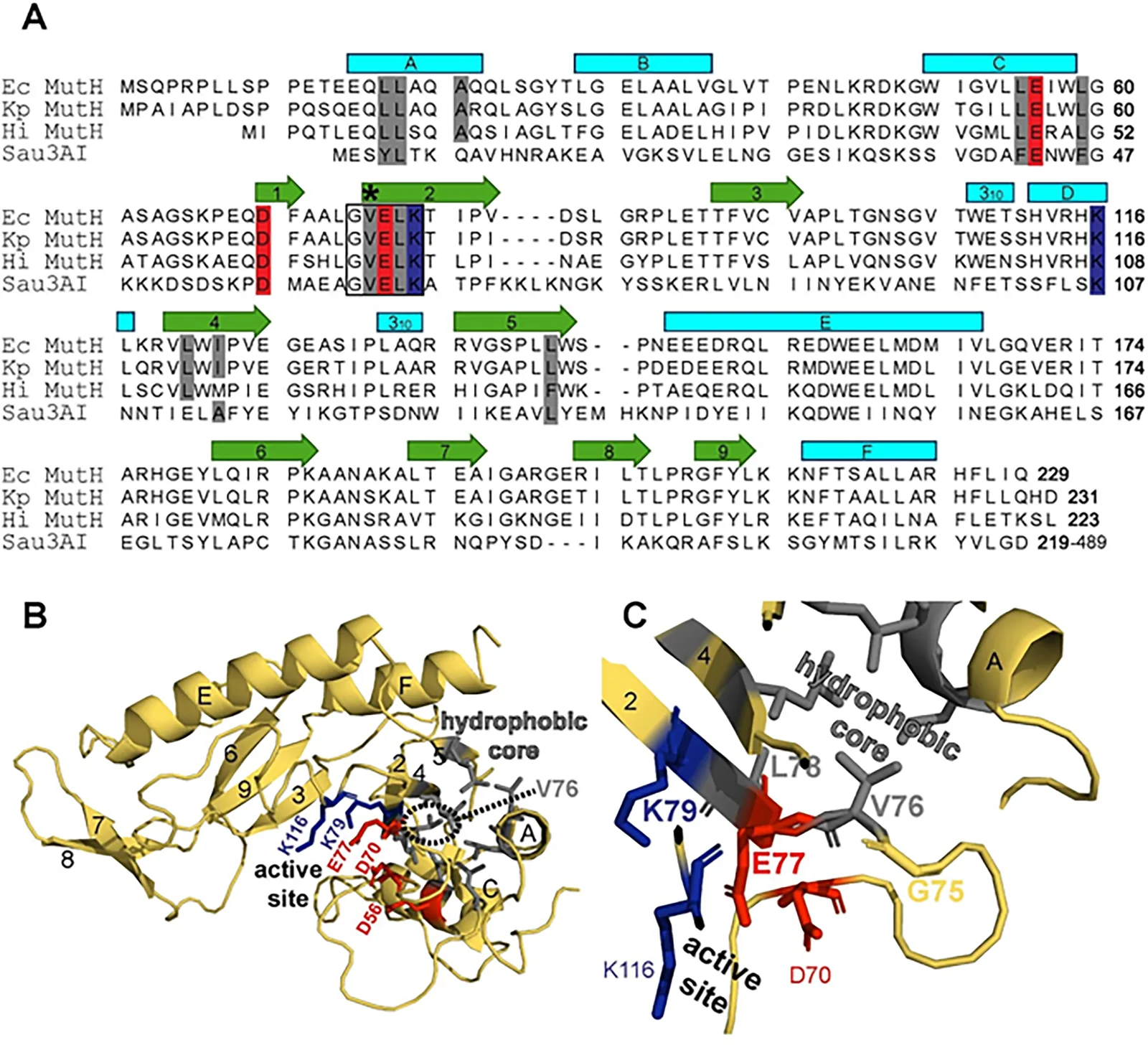
Structural analysis of V76G substitution on MutH. (**A**) Sequence alignment of the MutH family, including MutH from *Escherichia coli* (Ec), *Klebsiella pneumoniae* (Kp), *Haemophilus influenzae* (Hi), and Sau3AI from *Staphylococcus aureus*. Secondary structure annotations in the alignment are per Ban and Yang (26). The conserved GVELK motif is boxed, and the position of residue V76 is indicated with an asterisk. Secondary structure elements from the *E. coli* MutH crystal structure are shown above the alignment, where cyan boxes denote α-helices and green arrows denote β-strands. (**B**) Ribbon diagram of the published MutH structure from *E. coli* (pdb: 2AZO) (26). (**C**) Region surrounding V76. Residues of interest are drawn as sticks. Active site residues are shaded red (acidic) or blue (basic). Residues comprising a hydrophobic core between α-helices A and C, and β-sheets 2, 4, and 5 are shaded gray. Residues 75–79 of the conserved GVELK motif (β-sheet 2) are bolded in panel C. The analysis shows that the nonpolar sidechain of V76 points into a hydrophobic core, which likely assists in stabilizing the relative positioning of the neighboring active site residues to allow for catalysis. V76G substitution is expected to disrupt catalysis due to loss of this stabilizing hydrophobic interaction.

*K. pneumoniae* MutH shares 82.5% sequence identity with *E. coli* MutH, for which a crystal structure has been solved (26). Alignment across MutH family members shows that V76 lies within a highly conserved five-residue GVELK motif that contains two active-site residues (E77, K79) and two residues (V76, L78) that stabilize the hydrophobic core of the N-terminal subdomain (Fig. 3) (26). Substitution of valine with glycine at this position is therefore predicted to destabilize the active site environment and impair MutH function.

**Fig 4:**
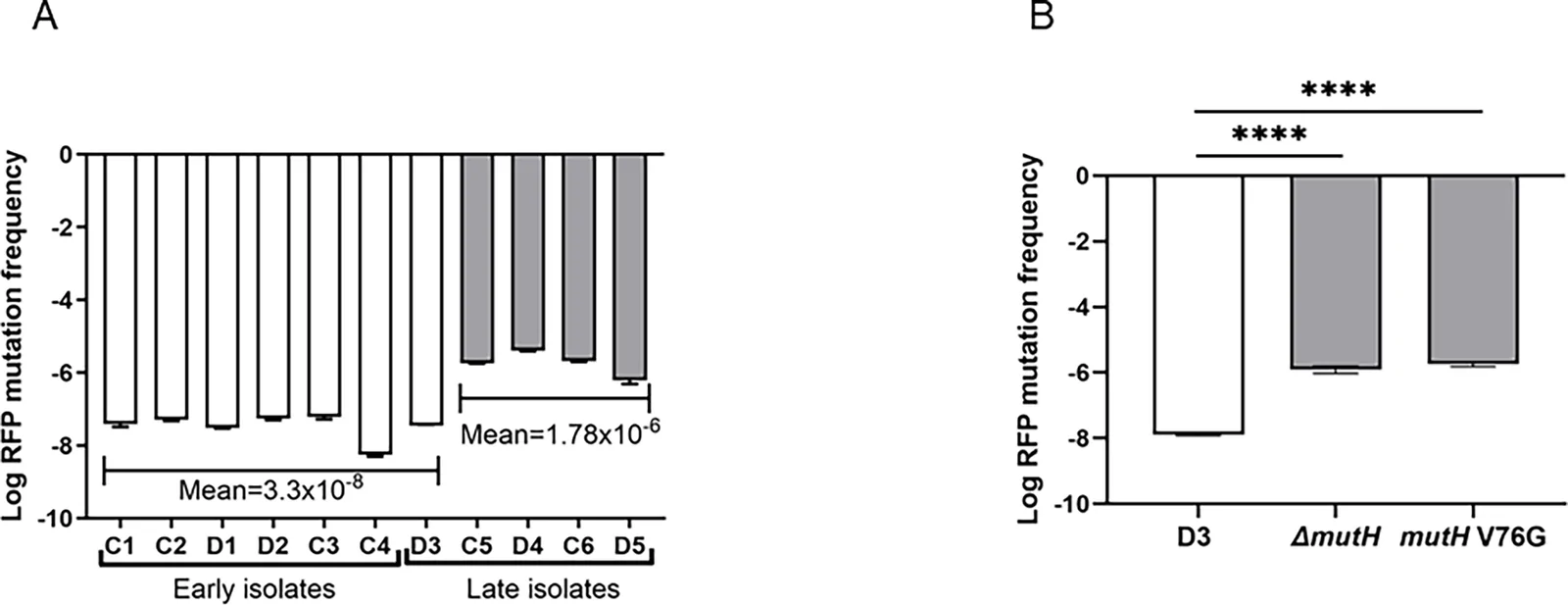
Evaluation of the contribution of *mutH* to rifampin mutational frequency (RFP). Panel **A** shows RFPs of clinical isolates, and panel **B** shows results from engineered isogenic strains differing only in the *mutH* allele (wild type, Δ*mutH*, and *mutH*^V76G^). Engineered mutants were generated from *mutH* wild-type isolate D3 using a *Klebsiella*-specific CRISPR–Cas9 platform. Data represent three independent experiments. (**A**) Log₁₀ RFP of 11 longitudinal clinical isolates. *mutH* mutant isolates exhibited significantly higher log₁₀ RFP than wild-type isolates (*P* < 0.0001). White and gray bars denote *mutH* wild-type and mutant isolates, respectively. (**B**) Log_10_ RFP of engineered isogenic strains. Both engineered mutants demonstrated higher RFP than the D3 parent strain (Δ*mutH*, –5.91 ± 0.12; *mutH*^V76G^, –5.74 ± 0.08; vs D3, –7.89 ± 0.03; *P* = 0.002 and 0.001, respectively; two-way ANOVA with Sidak’s multiple comparisons test).

To assess the functional impact of this substitution, we examined the hypermutator phenotype associated with *mutH*^V76G^. Clinical isolates carrying *mutH*^V76G^ exhibited significantly elevated rifampin mutational frequencies (RFP) *in vitro* compared with wild-type *mutH* isolates (log_10_ mean ± standard error of the mean (SEM): −5.75 ± 0.09 vs −7.48 ± 0.08, *P* < 0.00001) (Fig. 4A), exceeding the typical hypermutation threshold (RFP >10⁻⁶) (24).

**Fig 5:**
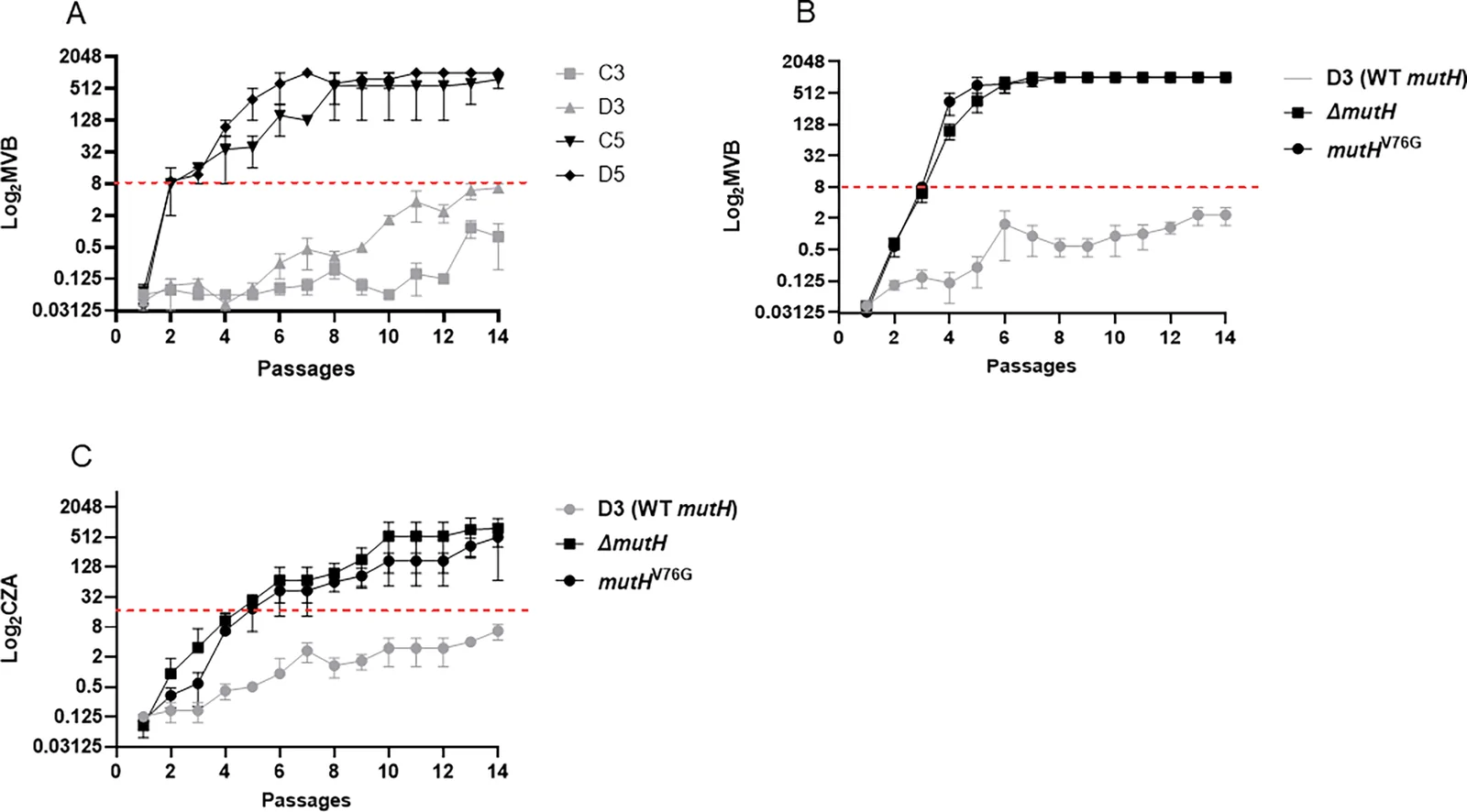
Impact of *mutH* on the evolution of antibiotic resistance during serial passage in meropenem-vaborbactam (MVB) and ceftazidime-avibactam (CZA) *in vitro*. Fourteen serial passages were performed using escalating concentrations of MVB (Fig. 5A) or CZA (Fig. 5B). For MVB, meropenem (0.03–1,024 µg/mL) was combined with a fixed vaborbactam concentration of 8 µg/mL; for CZA, ceftazidime (0.03–1,024 µg/mL) was combined with fixed avibactam at 4 µg/mL. CLSI nonsusceptibility breakpoints for MVB (≥8/8 µg/mL) and CZA (≥16/4 µg/mL) are indicated by red dashed lines. Data are from three independent experiments. (**A**) Serial passage of four clinical isolates in MVB, two of which harbored wild-type *mutH* (C3, D3), and two of which harbored *mutH*^V76G^ (C5, D5). Mutant isolates developed MVB resistance by passage 3, with MICs exceeding 256 µg/mL by passage 8, whereas wild-type isolates (C3, D3) remained susceptible through all 14 passages. (**B**) Serial passage of *mutH* wild-type isolate D3 and two engineered mutants (Δ*mutH* and *mutH*^V76G^) in MVB. Engineered *mutH* mutants exhibited more rapid increases in MVB MICs than the D3 parent strain, achieving resistance by passage 3. (**C**) Serial passage of engineered *mutH* mutants in CZA. *mutH* mutants developed elevated CZA MICs more rapidly than wild-type D3 and became resistant after five passages. CZA passage experiments were not performed for clinical isolates because *mutH*^V76G^ isolates were already CZA-resistant.

In 14-day serial passage experiments with escalating MVB concentrations *in vitro*, representative *mutH*^V76G^ isolates (C5, D5) developed MVB resistance by passage 3 and attained MICs >256 µg/mL by passage 8. In contrast, representative *mutH* wild-type strains (C3, D3) remained susceptible after 14 passages (Fig. 5A).

### Isogenic mutants confirm hypermutation, accelerated resistance evolution and increased plasmid transfer efficiency

We generated isogenic Δ*mutH* (*mutH* gene disruption) and *mutH*^V76G^ strains from isolate D3 using a *Klebsiella*-specific CRISPR-Cas9 (27). WGS confirmed no off-target effects (28). Δ*mutH* and *mutH*^V76G^ mutants exhibited 106-fold and 157-fold higher RFP than D3 (mean log_10_: −5.91 ± 0.12 and −5.74 ± 0.08, respectively, vs −7.94 ± 0.02) (Fig. 4B). To quantify mutation rates, we also performed fluctuation analysis. Both Δ*mutH* and *mutH*^V76G^ strains exhibited >200-fold increases in mutation rate relative to wild-type strain D3, confirming a hypermutator phenotype (Fig. S2).

In MVB passage experiments, mutant strains became MVB resistant after passage 3, while D3 remained susceptible through passage 14 (Fig. 5B). In escalating CZA concentrations, mutant strains developed CZA resistance by passage 5, while D3 remained susceptible through passage 14 (Fig. 5C).

**Fig 6:**
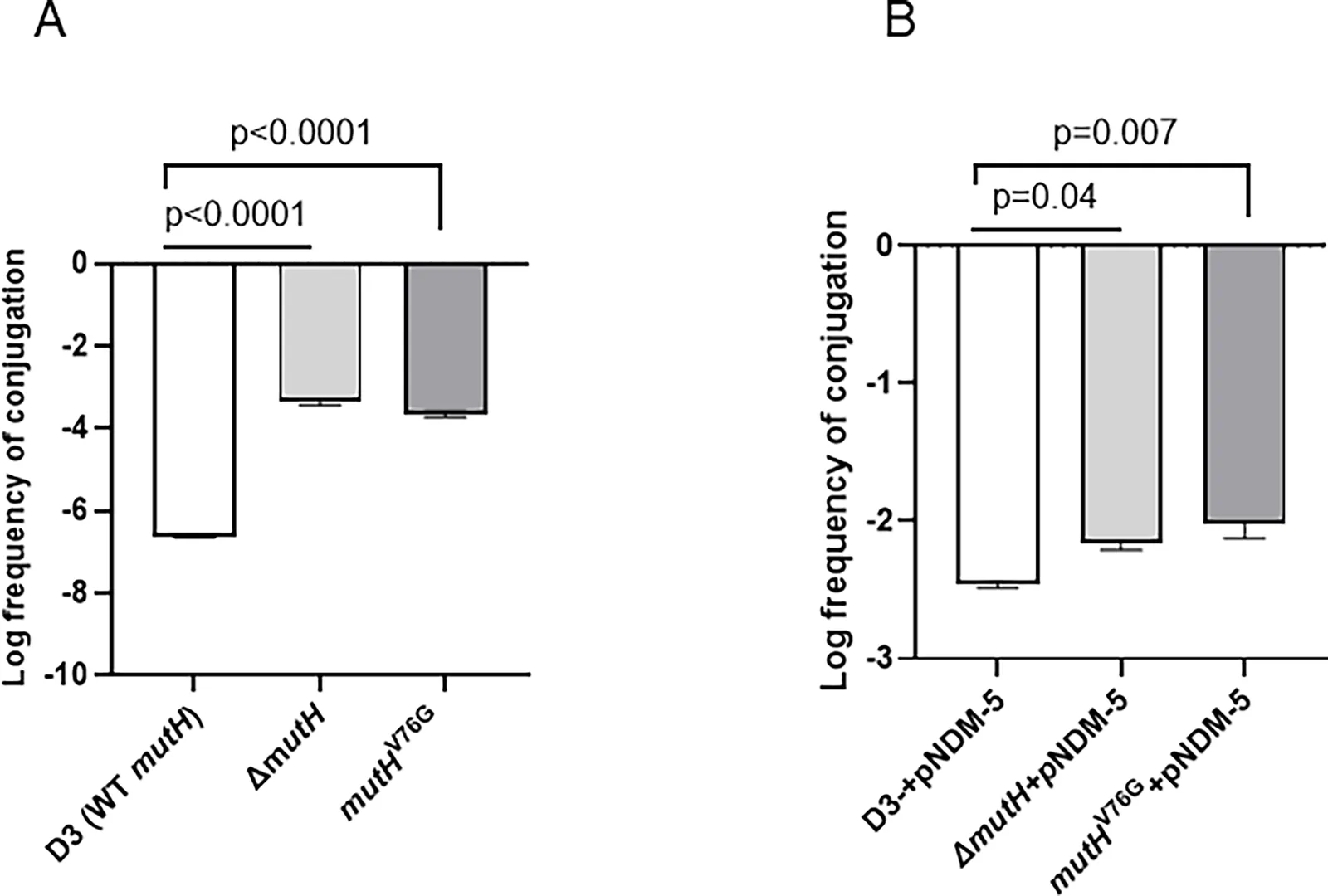
*mutH* impact on conjugation frequency. Conjugation frequencies were calculated based on recovery of recipient strain transconjugants, reflecting efficiency of plasmid transfer from donor strains. (**A**) Conjugation frequency for recipient *K. pneumoniae* strains harboring wild-type or engineered mutant *mutH*. The donor was *E. coli* clinical isolate XDR-74 carrying the IncX3 plasmid with *bla*_NDM-5_. Conjugation frequencies of Δ*mutH* (log_10_ –3.36 ± 0.08) and *mutH*^V76G^ (log_10_ –3.65 ± 0.08) isogenic strains were significantly higher than that of wild-type *mutH* parent isolate D3 (log_10_ –6.62 ± 0.02; *P* < 0.0001 for both comparisons, one-way ANOVA with Dunnett’s multiple comparisons test). (**B**) Conjugation frequency for donor *K. pneumoniae* strains harboring wild-type (isolate D3) or mutant *mutH* (Δ*mutH*, *mutH^V76G^*). Donor strains carried the IncX3 plasmid with *bla*_NDM-5_. The recipient was *E. coli* J53 carrying pCasApr. Δ*mutH* (log_10_ –2.16 ± 0.05) and *mutH^V76G^* (log_10_ –2.02 ± 0.10) strains exhibited significantly higher conjugation frequency than did wild-type *mutH* parent strain D3 as donor (log_10_ –2.46 ± 0.28; *P* = 0.04 and *P* = 0.007, respectively; one-way ANOVA with Dunnett’s multiple comparisons test).

To study conjugative transfer, we first used *E. coli* XDR-74 carrying an IncX3 *bla*_NDM-5_ (Fig. S3) as the donor strain and engineered Δ*mutH* and *mutH*^V76G^ mutants and D3 as recipients (29). Acquisition of IncX3 with *bla_NDM-5_* by Δ*mutH* and *mutH*^V76G^ mutants was significantly greater than by D3 (conjugation frequencies ~ 1,820-fold [3.36-log] and 930-fold [2.97-log] higher, respectively; *P* < 0.0001) (Fig. 6A). When *E. coli* J53-pCasApr served as recipient and Δ*mutH*, *mutH*^V76G^, or D3 carrying blaNDM-5–IncX3 served as donors, Δ*mutH* and *mutH*^V76G^ demonstrated modest but significant increases in donor efficiency (~2-fold and ~2.75-fold, respectively; *P* = 0.04 and 0.01) (Fig. 6B).

### *mutH*^V76G^ enhances fitness and accelerates resistance *in vivo*

**Fig 7:**
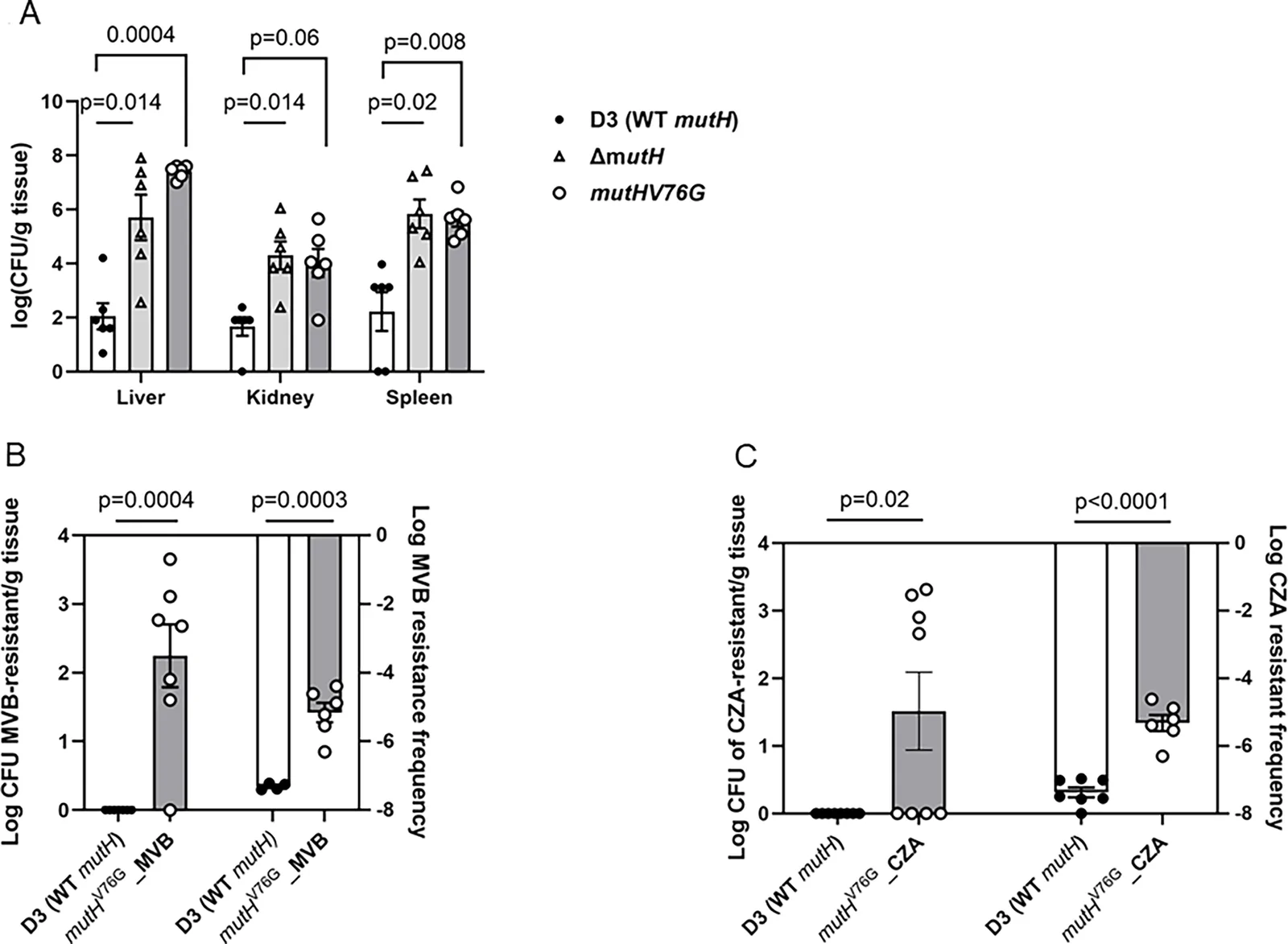
*mutH*^V76G^ impact on *K. pneumoniae* fitness *in vivo* and emergence of resistance during MVB and CZA treatment. (**A**) *mutH* impact on strain fitness. Mice were infected intravenously with 5 × 10⁴ CFU/mouse of wild-type *mutH* isolate D3 or engineered *mutH* mutant strains (Δ*mutH* or *mutH*^V76G^). Mice were sacrificed on day 3, and bacterial burdens were quantified in livers, kidneys, and spleens. In livers, mean ± SEM log_10_ CFU/g was 2.05 ± 0.48 (D3), 5.70 ± 0.84 (Δ*mutH*, *P* = 0.014) and 7.40 ± 0.210 (*mutH*^V76G^, *P* = 0.0004). In kidneys, mean burdens were 1.66 ± 0.34 (D3), 4.30 ± 0.54 (Δ*mutH*, *P* = 0.014) and 4.02 ± 0.51 (*mutH*^V76G^, *P* = 0.06) log_10_ CFU/g. In spleens, mean burdens were 2.22 ± 0.72 (D3), 5.84 ± 0.53 (Δ*mutH*, *P*=0.02), and 5.64 ±  0.28 (*mutH^V76G^*, *P* = 0.008) log_10_ CFU/g. Statistical analysis was performed using two-way ANOVA with Turkey’s multiple comparisons test. (**B and C**) *mutH* impact on emergence of MVB or CZA resistance. Mice were infected intravenously with 1 × 10^5^ CFU/mouse of isolate D3 or the isogenic *mutH^V76G^* strain. The *mutH^V76G^* strain was chosen since this mutation rather than Δ*mutH arose in clinical isolates.* MVB and CZA MICs against D3 and the *mutH^V76G^* strain were 0.125 and 0.25 µg/mL, respectively. Mice were treated with MVB (50 mg/kg, intraperitoneal, every 8 h) or CZA (37.5 mg/kg intraperitoneal every 8 h) starting 4 h post-infection. On day 3, mice were sacrificed, and bacterial burdens in the liver, kidney, and spleen were quantified. Homogenates were plated on LB agar with and without 4 × MIC of the respective antibiotic. Log_10_ resistance frequency indicates the proportion of recovered bacteria within a population and is calculated as: log_10_ (bacterial count on 4× MIC plates/bacterial count on no antibiotic plates). (**B**) Log_10_ CFU/g of strains in tissues from MVB treated mice, as identified by growth on plates containing 4× MIC MVB. Strains were recovered exclusively from the livers of mice infected with the *mutH^V76G^* mutant (2.24 ± 0.46 log_10_ CFU/g enumerated on MVB-containing plates). No strains were isolated from MVB treated D3-infected mice. Log_10_ resistance frequencies for D3 and *mutH^V76G^* were –7.31 ± 0.05 and –5.1 ± 0.28, respectively (*P* = 0.0003). All isolates recovered from MVB-containing plates were confirmed to have MVB MICs ≥ 16 µg/mL. (7C) Log_10_ CFU/g of strains in tissues from CZA treated mice, as identified by growth on plates containing 4× MIC CZA. Strains were recovered exclusively from the livers of mice infected with the *mutH^V76G^* mutant (1.51 ± 0.24 log_10_ CFU/g enumerated on CZA-containing plates). No strains were isolated from CZA treated D3-infected mice. Log_10_ recovery frequencies for D3 and *mutH^V76G^* were –7.37 ± 0.15 and –5.33 ± 0.24, respectively (*P* < 0.0001). Among 16 randomly selected isolates from CZA-containing plates, seven were confirmed to have CZA MICs ≥ 16 µg/mL.

We immunocompromised CD1 mice (six per group) with cyclophosphamide and cortisone, then intravenously infected them with engineered Δ*mutH*, *mutH*^V76G^, or D3 (5x10⁴ CFU/mouse). On day 3, bacterial burdens of Δ*mutH* were significantly higher than those of D3 in all organs (liver, *P* = 0.014; kidney, *P* = 0.014; spleen, *P* = 0.02) (Fig. 7A). Burdens of the *mutH*^V76G strain^ were significantly higher than those of D3 in liver (*P* = 0.0004) and spleen (*P* = 0.008). Burdens of Δ*mutH* and *mutH*^V76G^ did not differ significantly in any organ.

In separate experiments, mice infected with *mutH*^V76G^ strain or D3 (1 × 10^5^ CFU/mouse) were treated with MVB or CZA beginning 4 h post-infection. No colonies were recovered from D3-infected mice on plates containing 4× MIC antibiotic. In contrast, *mutH*^V76G^-infected mice yielded recoverable colonies (mean log10 CFU/g ± SEM: 2.24 ± 0.46 for MVB; 1.51 ± 0.24 for CZA). Corresponding recovery frequencies were significantly higher for *mutH*^V76G^ vs D3 (MVB: –5.16 ± 0.28 vs –7.31 ± 0.05, *P* = 0.0003; CZA: –5.33 ± 0.24 vs –7.37 ± 0.15, *P* < 0.0001) (Fig. 7B and C). Corresponding MVB recovery frequencies of *mutH*^V76G^ and D3 were log_10_ –5.16 ± 0.28 and –7.31 ± 0.05 (*P* = 0.0003) (Fig. 7B). CZA recovery frequencies were log_10_ –5.33 ± 0.24 and −7.37 ± 0.15, respectively (*P* < 0.0001). (Fig. 7C).

Sequencing of MVB-resistant isolates revealed *ompK36* loss-of-function mutations (Q76STOP, Q92STOP, L361P, L363P) with reduced transcript and protein levels as assessed by qRT-PCR and SDS-PAGE, respectively. (Fig. S4). CZA-resistant isolates carried *bla*_KPC-3_ variants (A172T, R164H) previously associated with CZA resistance (30, 31).

## DISCUSSION

Multidrug-resistant *K. pneumoniae*, in particular CRKP, poses formidable clinical challenges, which have been exacerbated by emergence of resistance to newly developed “last-line” agents like ceftazidime–avibactam (CZA) and meropenem–vaborbactam (MVB). In this study, we tracked 11 KPC-producing *K. pneumoniae* isolates collected over ~4 years, providing a detailed longitudinal view of within-host evolution. We identified a single nucleotide change in the DNA mismatch repair gene *mutH*, resulting in a V76G substitution (*mutH*^V76G^) that converted the strain into a hypermutator. *mut*^V76G^ drove rapid accumulation of genome-wide SNPs, facilitated the emergence of CZA resistance via KPC Ω-loop substitutions, altered OmpK36 porin, and caused stepwise reductions in susceptibility to MVB and cefiderocol (FDC). *mutH*^V76G^ also enhanced horizontal acquisition of resistance-conferring plasmids, amplifying the bacterium’s ability to gain extrinsic resistance determinants. Together, these findings reveal how a single mutation in *mutH* can accelerate multiple adaptive pathways, posing a substantial threat to the efficacy of currently available antibiotics.

Functional defects in Mut proteins accelerate genome-wide mutation, impacting antibiotic resistance, stress responses, and other adaptive traits (32). Hypermutator phenotypes have been reported infrequently among ST258 CRKP. However, their prevalence is likely underestimated because hypermutation is infrequently assessed, most studies examine single isolates, and research often focuses on acute infections rather than chronic colonization or recurrent infections, where mutational frequency is higher (11, 22, 23, 25, 32–34).

*mutH*^V76G^ variants have not been described previously in any species. Structural comparisons to other MutH family members suggest a plausible mechanism for loss of function (35). MutH consists of N- and C-terminal subdomains, with the active site located in the N-arm. The nonpolar side chain of V76 resides within the hydrophobic core of the N-arm, stabilizing neighboring active site residues critical for metal ion coordination and catalysis. Substitution of valine with glycine likely disrupted this stabilizing interaction, impairing endonuclease activity and converting the strain into a hypermutator. This mutation drove rapid accumulation of genome-wide SNPs, including KPC Ω-loop substitutions, OmpK36 alterations, and stepwise reductions in susceptibility to CZA, MVB, and FDC. Observations from clinical isolates were validated using an engineered isogenic *mutH*^V76G^ strain, which exhibited markedly accelerated resistance evolution under antibiotic pressure. Compared with the parent D3 isolate, the mutant showed increased mutational frequency and rapidly acquired resistance to MVB and CZA during serial *in vitro* passage and invasive mouse infections. MVB-treated mice accumulated ompK36 mutations (Q76STOP, L361P, L363P) that truncate or narrow the porin channel, whereas CZA-treated mice developed KPC Ω-loop substitutions (A172T, R164H). In the absence of antibiotics, *mutH*^V76G^ strains exhibited higher tissue burdens in mouse livers and kidneys then D3. Furthermore, *mutH*^V76G^ enhanced horizontal gene transfer, showing a ~3-log increase in acquisition of an IncX3 plasmid carrying *blaNDM-5* from *E. coli*, while modestly increasing plasmid donation (~2-fold). These results confirm that the V76G substitution abrogates MutH function, accelerates intrinsic resistance evolution, increases fitness *in vivo*, and amplifies acquisition of extrinsic resistance determinants.

The first seven longitudinal isolates, collected over ~3.3 years, were wild-type *mutH* and highly clonal, differing by only 2–15 SNPs, consistent with reported within-host evolution rates (7, 35, 36). Following a 2-week CZA course for bacteremia caused by the seventh isolate (D3), *mutH*^V76G^ hypermutator isolate C5 emerged, differing from prior isolates by 122–132 SNPs, far exceeding the expected evolutionary rate, and exhibiting a mutational spectrum consistent with MMR deficiency. The three subsequent isolates, collected over 21 weeks, also carried *mutH*^V76G^ and displayed progressive divergence (69–264 SNPs), while retaining the ST258 background, *bla*_KPC-3_ backbone, and conserved chromosomal and plasmid features. This abrupt increase in genome-wide SNPs coinciding with *mutH* inactivation supports a single hypermutator lineage driving rapid within-host evolution (22, 33). Temporal succession of isolates, correlations with antibiotic exposure, MICs, and resistance-conferring mutations further support this model, although acquisition of a highly related ST258 strain cannot be fully excluded.

Serial changes in antibiotic MICs among *mutH*^V76G^-carrying clinical isolates corresponded to specific emergent mutations. CZA resistance first appeared in isolate C5, accompanied by reversion from carbapenem resistance to susceptibility, mediated by the *bla*_KPC-3_^L169P^ substitution. This mutation, which persisted in D4 and C6, enhances ceftazidime hydrolysis, disrupts avibactam binding, and attenuates carbapenemase activity (36, 37). The final isolate (D5), recovered following MEM treatment for bacterial pneumonia, reverted to carbapenem resistance and CZA susceptibility, likely due to loss of *bla*_KPC-3_^L169P^ and acquisition of *bla*_KPC-3_^T243A^ substitution outside the Ω-loop. Mutations of T243 have been associated with increased carbapenem MIC, but they have inconsistent impact on CZA susceptibility (38, 39). D5 also carried an *ompK36*^Q301W^ substitution, which may contribute to carbapenem resistance, although its functional impact is untested. Susceptibility to FDC, a siderophore cephalosporin structurally related to ceftazidime, was also reduced in CZA-resistant isolates. Cross-resistance to CZA and FDC has been associated with combinations of KPC variants, porin alterations, and mutations in EnvZ or CirA, which may influence β-lactamase activity, porin function, and drug influx/efflux (40–42); the specific contributions of these mutations were not experimentally confirmed here

Limitations of this study include the focus on a single patient, which may limit generalizability, and the lack of experimental validation for some specific mutations. Despite these constraints, the study offers important mechanistic insights into hypermutation, and its contributions to rapid adaptation, multidrug resistance, and enhanced genetic exchange. The longitudinal, multi-isolate sampling over ~4 years, integrated with isogenic mutant construction and *in vivo* experiments, enabled us to directly attribute the *mutH*^V76G^ substitution to hypermutation, accelerated resistance development, and increased plasmid transfer. This study also provides a rare, high-resolution view of long-term, within-host evolution of *K. pneumoniae* under real-world selective pressures. Future studies should systematically define the prevalence and impact of hypermutator strains of CRKP and other CRE, screen multiple isolates per patient, identify mutations in *mut* and other MMR-related genes, and link genomic features with treatment outcomes.

## MATERIALS AND METHODS

### Clinical isolates, strains, and antimicrobial susceptibility testing

Clinical isolates, bacterial strains and plasmids used in this study are listed in Table S1. Unless otherwise specified, bacteria were cultured in 4 mL of Mueller–Hinton (MH) broth at 37°C with shaking at 180 rpm. Eleven *Klebsiella pneumoniae* isolates were collected from an immunocompromised patient enrolled in the CREST study (CRE and MDR-E carriage among solid organ transplant recipients) (7), which was approved by the University of Pittsburgh Institutional Review Board. Both colonizing- and disease-causing isolates were preserved at –80°C. *E. coli* XDR-74 carrying an IncX3 plasmid with bla_NDM-5_ was obtained from the UPMC XDR Pathogen laboratory. Species identification was performed by MALDI-TOF MS (Bruker). Antimicrobial susceptibility testing was conducted by broth microdilution according to CLSI guidelines (43), and MICs were interpreted using CLSI or FDA breakpoints.

### Whole-genome sequencing and phylogenetic analysis

Genomic DNA was extracted from overnight cultures using the Wizard Genomic DNA Purification Kit (Promega, Madison, WI). Sequencing libraries were prepared with the Nextera DNA Library Prep Kit and sequenced on an Illumina HiSeq platform (150 bp paired-end reads). Reads were quality-trimmed with Trimmomatic v0.39 and assembled *de novo* using SPAdes v3.15. Genome annotation was performed with Prokka. Multi-locus sequence typing and resistance gene detection were conducted with AMRFinderPlus v3.9 and Kleborate v2.0.1. Comparative genomics of ST258 *K. pneumoniae* isolates, including paired colonizing and disease-causing multidrug-resistant isolates, was performed using Snippy v4.6 with the earliest isolate as the reference. For core genome SNP analysis, variants within repetitive regions (identified with nucmer from MUMmer) or prophage regions (PHASTER) were excluded. Maximum-likelihood phylogenetic trees were generated with IQ-TREE (GTR+G model, 1,000 bootstrap replicates). Pairwise SNP distances were visualized using the *pheatmap* package in R.

### Rifampin mutation frequency assays

Hypermutability was assessed by plating serial dilutions (~10^10^ CFU/mL) on rifampin-containing (300 µg/mL) and drug-free MH agar (44). Mutation frequency was calculated as the ratio of colonies on rifampin plates to those on drug-free plates. Each isolate was tested in triplicate, and the mean ± SEM mutation frequency was reported.

### Mutation rates

Rates were determined using the Jones protocol (45), a variant of the traditional fluctuation assay. For each strain, five replicate flasks, each containing 25 mL LB broth, were inoculated with ~100 CFU and cultured to saturation at 37°C overnight with shaking. To determine total population size, dilutions were plated on LB agar and, to score mutants, LB agar containing 100 μg/mL rifampin. Plates were incubated at 37°C for 24 h prior to CFU counting. Mutation rates and 95% credible intervals were estimated using the method of maximum likelihood.

### *In vitro* serial passage

Serial passaging was performed as described, with modifications (46). Briefly, experiments were carried out in 96-well plates with twofold serial dilutions of antibiotics in Mueller–Hinton (MH) broth. Ceftazidime and MEM were tested at concentrations ranging from 0.03 to 1,024 µg/mL with fixed 4 µg/mL avibactam or 8 µg/mL vaborbactam concentration, respectively. Each well was inoculated with ~4 × 10⁶ cells, incubated aerobically at 35°C for 18–24 h, and growth (OD₆₀₀ > 0.2) from the highest antibiotic concentration was diluted 1:100 into fresh medium. This process was repeated daily for 14 passages. MICs were determined every two passages by broth microdilution.

### Construction of *ΔmutH* mutant

A *mutH* deletion mutant was generated using a CRISPR/Cas9-mediated genome editing system (27). The plasmid pCasKP-apr was electroporated into strain D3 and selected on LB agar containing 30 µg/mL apramycin, followed by PCR confirmation. An sgRNA targeting *mutH* was designed using the SSC online server and cloned into pSGKP-Rif, yielding pSGKP-mutH-N20. A 413-bp deletion repair template was amplified by two-step PCR using primers mutH-A/B/C/D. The repair DNA and pSGKP-mutH-N20 were co-transformed into L-arabinose-induced, pCasKP-apr-positive D3 cells. Cultures were plated on LB agar containing 30 µg/mL apramycin and 100 µg/mL rifampin, and candidate mutants were screened by PCR and confirmed by sequencing. To cure editing plasmids, mutants were grown in antibiotic-free LB broth and streaked on LB agar with 5% sucrose; colonies retaining the deletion but lacking both pCasKP-apr and pSGKP-RFP were confirmed by the inability to grow on antibiotic-containing plates.

### Allelic replacement of *mutH* with *mutH*^V76G^

*mutH*^V76G^ along with ~500  bp of flanking upstream and downstream regions was amplified from isolate D5 DNA using primers KpMutH-complement-For and KpMutH-complement-Rev (Table S2). The PCR product was cloned into the *Not*I site of the pKOV vector and verified by Sanger sequencing. The resulting recombinant plasmid, pKOV-*mutH*-V76G, was co-electroporated with pSGKP-mutH-N20 into the L-arabinose-induced *D3* isolate harboring the pCasKP-apr plasmid. Successful allelic replacement of native *mutH* locus with the *mutH*^V76G^ variant was confirmed by PCR and WGS. Plasmids were cured as described above.

### Conjugation assays

Conjugation experiments were performed using a broth mating protocol adapted from Huang et al. (29), with minor modifications. Donor and recipient strains were grown in Mueller–Hinton (MH) broth with appropriate antibiotics at 37°C to mid-exponential phase, washed three times with fresh MH, and adjusted to OD₆₀₀ ~0.5. Equal volumes (0.5 mL) of donor and recipient cultures were mixed (1:1), pelleted, resuspended in 50 µL MH, and 25 µL was spotted onto 0.45-µm membrane filters on MH agar. Plates were incubated overnight at 37°C. After incubation, cells were recovered, serially diluted, and plated on CHROMagar with selective antibiotics. Controls included donor and recipient strains plated separately. Plasmid uptake (“recipient” assay)*:* Recipient isolate D3 (WT *mutH*) and Δ*mutH*, and *mutH*^V76G^ mutant strains were mated with *E. coli* XDR-74 carrying IncX3-*NDM-5* and selected with 50 mg/L kanamycin, and 16 mg/L ceftazidime with 4 mg/L avibactam. Plasmid donation (“donor” assay)*:* Donor strains D3::IncX3-*NDM-5*, Δ*mutH*::IncX3-*NDM-5*, and *mutH* V76G::IncX3-*NDM-5* were mated with *E. coli* J53::pCasApr (apramycin resistance) and selected with 100 mg/L apramycin, and 16 mg/L ceftazidime with 4 mg/L avibactam. Plates were incubated at 37°C for 18–24 h and colonies were enumerated, and the presence of *NDM* was confirmed by PCR. Conjugation frequency was calculated as the number of transconjugants per donor. All experiments were performed in triplicate.

### Mouse model of disseminated *K. pneumoniae* infection

Male and female ICR CD1 mice (20–25 g, Envigo) were used in equal number. Transient immunosuppression was induced via intraperitoneal cyclophosphamide (150 mg/kg 4 days prior and 100 mg/kg 1 day prior to infection) and subcutaneous cortisone (20 mg/kg 4 days prior and 1 day prior). Mice were intravenously inoculated via the lateral tail vein with 5 × 10⁴ (tissue burden studies) or 1 × 10^5^ (emergence of resistance during MVB and CZA treatment studies) CFU of *K. pneumoniae*. For tissue burden tissue studies, mice were sacrificed on day 3, and the liver, kidney, and spleen were collected for colony enumeration. For emergence of resistance studies, the mice were treated with MVB (50 mg/kg intraperitoneally every 8 h) or CZA (37.5 mg/kg intraperitoneally every 8 h) starting at 4 h post-infection. On day 3, mice were sacrificed, and the liver, kidney, and spleen were homogenized and plated on LB agar with and without 4× MIC to screen for resistant isolates (CZA concentration: 1 µg/mL ceftazidime with 4 µg/mL avibactam; MVB concentration: 0.5 µg/mL MEM with 8 µg/mL vaborbactam). Tissue burdens were log_10_-transformed and expressed as mean ± SEM log_10_ CFU/gram of tissue. The difference between groups was determined by Wilcoxon test. *P-*value <0.05 was considered statistically significant.

### Statistical analysis

Data were analyzed using GraphPad Prism, v10.6.0 (GraphPad Software). When appropriate, data were log-transformed. Data were presented as mean ± SEM. Continuous variables were compared using Mann-Whitney U or Wilcoxon tests. For comparison of >2 groups, one-way ANOVA with Dunnett’s multiple comparisons test was used. Statistical significance was defined as *P-*value < 0.05 (two-tailed).

## Data Availability

The genomic sequencing data generated during this study have been deposited in Figshare and are publicly available at https://doi.org/10.6084/m9.figshare.33112382. The data have also been submitted to the NCBI Sequence Read Archive (SRA). The corresponding accession number will be added to this article once processing by NCBI is complete.

## SUPPLEMENTAL MATERIAL

Supplemental material aac.00189-26-s0001.docx Tables S1 and S2; Fig. S1 to S4.

ASM does not own the copyrights to Supplemental Material that may be linked to, or accessed through, an article. The authors have granted ASM a non-exclusive, world-wide license to publish the Supplemental Material files. Please contact the corresponding author directly for reuse.
