# Supplementary material for: Emergence of mutHV76G in carbapenem-resistant Klebsiella pneumoniae disrupts DNA mismatch repair and results in a hypermutator phenotype
Source: Antimicrob Agents Chemother. 2026 Aug 5;70(9):e00189-26. doi: 10.1128/aac.00189-26 (PMC13712981; doi:10.1128/aac.00189-26)
Supplement: Supplementary file 1 [file aac.00189-26-s0001.docx]

**Supplemental tables.**

**Table S1. Bacterial strains and plasmids used in this study.**

| **Bacteria** | **Sources** |
| --- | --- |
| Longitudinal ST258 *K. pneumoniae* isolates (*n=*11, described in Figure 1, Table 2) | This study |
| Clinical isolate *Escherichia coli* XDR-74 (IncX-NDM) | This study |
| *K. pneumoniae* Δ*mutH*, derived from isolate D3 | This study |
| *K. pneumoniae* *mutH*^V76G^, derived from isolate D3 | This study |
| *K. pneumoniae* Δ*mutH::IncX-NDM5*, derived from *K. pneumoniae* Δ*mutH* | This study |
| *K. pneumoniae* *mutH*^V76G^::*IncX-NDM5*, derived from *K. pneumoniae* *mutH*^V76G^ | This study |
| DH5α | New England BioLabs |
| *E. coli* J53 | Gibson TC et al ^47^ |
| *E.coli* J53::pCasKP-apr | This study |
| **Plasmids** | **Sources** |
| pCasKP-apr | Wang Y. et al. ^27^ |
| pSGKP-Rif | Hao M et al ^28^ |
| pKOV | Addgene |

Gibson TC, Scheppe ML, Cox EC. Fitness of an Escherichia coli mutator gene. Science 1970;169:686-8.

Wang Y, Wang S, Chen W, et al. CRISPR-Cas9 and CRISPR-Assisted Cytidine Deaminase Enable Precise and Efficient Genome Editing in Klebsiella pneumoniae. Appl Environ Microbiol 2018;84.

Hao M, He Y, Zhang H, et al. CRISPR-Cas9-Mediated Carbapenemase Gene and Plasmid Curing in Carbapenem-Resistant Enterobacteriaceae. Antimicrob Agents Chemother 2020;64.

**Table S2. Primers used in this study.**

| **Primer name** | **sequence 5'-3'** |
| --- | --- |
| *mutH*-Spacer1_For | TAGTCGAGCAGGATTTCGCCGCGCT |
| *mutH*-Spacer1_Rev | AAACAGCGCGGCGAAATCCTGCTCG |
| *mutH*-A | TTGTCAGTGACCAGTTCTGGA |
| *mutH*-B | AAATCCTGCTCGGGTTTACT |
| *mutH*-C | ATGGACTGGGAAGAGCTGATGAGTAAACCCGAGCAGGATTT |
| *mutH*-D | CCCCAACGCAGAACAAAAAA |
| p*MutH*-complement_For | ATAAGAATGCGGCCGCATGATTGTTTCACACTCGCC |
| *mutH*-complement_Rev | ATAAGAATGCGGCCGCATGACGGTCAACAAAATCGC |
| *mutH*seq_For | CAGTTCTGGAAAACCCGTTT |
| *mutH*seq_Rev | AGGATCGGTTATCCATGCAA |
| *mutH* SYBR2_For | TGGAGACCACCTTCGTCTG |
| *mutH* SYBR2_Rev | CTGCAGCTTATGCCGTACAT |
| *cirA_*For | TGCCGATCTTCCGCTATTT |
| *cirA_*Rev | GACCATCGTTGTAGGTGTAGTT |
| *envZ_*For | CTATGGCAATGGCTGGATCA |
| *envZ*_R | CCCGCTGCTCAGGTTTAAT |

**Supplemental figures.**

**Figure S1.** **Nucleotide and amino acid alignment of *mutH* and MutH from longitudinal *K. pneumoniae* isolates.**  Wild-type *mutH* and *mutH*^V76G^ are evident in isolates D3 and D5, respectively.

**Figure S2.** **Fluctuation-based mutation rate analysis of *mutH* mutants.** Mutation rates (mutations per cell per generation) and 95% credible intervals (error bars) were determined using fluctuation assays based on rifampin resistance, with rates estimated by the method of maximum likelihood. Five independent replicate cultures were analyzed for each strain. Both Δ*mutH* and *mutH*^V76G^ mutants exhibited substantially higher mutation rates than the wild-type parent strain, consistent with a hypermutator phenotype.

**Figure S3. Circular map of the donor IncX3 plasmid carrying *bla*NDM-5 used for conjugation experiments.**

**Figure S4. OmpK36 expression by isolate D3, the engineered isogenic *mutH*_V76G_ mutant and MVB-resistant isolates recovered from mice.** SDS-PAGE (12%) analysis of outer membrane proteins from wild-type parent isolate D3, isogenic *mutH*^V76G^, and four MVB-resistant isolates (#1, #9, #22, #27) recovered from livers of mice infected with *mutH*^V76G^. The prominent ~35 kDa band (denoted by black arrow) corresponds to OmpK36. Protein levels correlate with ompK36 expression quantified by qRT-PCR (values shown below lanes, normalized to *mutH*^V76G^). Decreased protein and mRNA expression was observed in resistant isolates compared to parental strains. Protein ladder: PageRuler™ Prestained Protein Ladder (Thermo Scientific).
